# Image reconstruction impacts haemodynamic parameters derived from 4D flow magnetic resonance imaging with compressed sensing

**DOI:** 10.1093/ehjimp/qyae137

**Published:** 2024-12-17

**Authors:** Pia Sjöberg, Tania Lala, Johan Wittgren, Ning Jin, Erik Hedström, Johannes Töger

**Affiliations:** Clinical Physiology, Department of Clinical Sciences Lund, Lund University, Lund 221 00, Sweden; Department of Clinical Physiology, Skåne University Hospital, Lund 221 85, Sweden; Clinical Physiology, Department of Clinical Sciences Lund, Lund University, Lund 221 00, Sweden; Biomedical Engineering, Lund University, Lund, Sweden; Clinical Physiology, Department of Clinical Sciences Lund, Lund University, Lund 221 00, Sweden; Department of Clinical Physiology, Skåne University Hospital, Lund 221 85, Sweden; Cardiovascular MR R&D, Siemens Medical Solutions USA, Inc., Cleveland, OH, USA; Clinical Physiology, Department of Clinical Sciences Lund, Lund University, Lund 221 00, Sweden; Department of Clinical Physiology, Skåne University Hospital, Lund 221 85, Sweden; Diagnostic Radiology, Department of Clinical Sciences Lund, Lund University, Lund, Sweden; Department of Radiology, Skåne University Hospital, Lund, Sweden; Clinical Physiology, Department of Clinical Sciences Lund, Lund University, Lund 221 00, Sweden; Department of Clinical Physiology, Skåne University Hospital, Lund 221 85, Sweden; Biomedical Engineering, Lund University, Lund, Sweden

**Keywords:** cardiac magnetic resonance imaging (CMR), congenital heart disease, paediatric, haemodynamic forces, kinetic energy

## Abstract

**Aims:**

4D blood flow measurements by cardiac magnetic resonance imaging (CMR) can be used to simplify blood flow assessment. Compressed sensing (CS) can provide better flow measurements than conventional parallel imaging (PI), but clinical validation is needed. This study aimed to validate stroke volume (SV) measurements by 4D-CS in healthy volunteers and patients while also investigating the influence of the CS image reconstruction parameter *λ* on haemodynamic parameters.

**Methods and results:**

Healthy participants (*n* = 9; 20–62 years) underwent CMR with 2D, 4D-CS, and 4D-PI flow. Patients (*n* = 30, 17 with congenital heart defect; 2–75 years) had 4D-CS added to their clinical examination. Impact of *λ* was assessed by reconstructing 4D-CS data for six different *λ* values. In healthy volunteers, 4D-CS and 4D-PI SV differed by 0.4 ± 6.5 mL [0.6 ± 9.1%; intraclass correlation coefficient (ICC) 0.98], and 4D-CS and 2D flow by 0.9 ± 7.0 mL (0.9 ± 10.6%; ICC 0.98). In patients, 4D-CS and 2D flow differed by −1.3 ± 6.0 mL (−7.2 ± 20%; ICC 0.97). SV was not dependent on *λ* in patients (*P* = 0.75) but an increase in *λ* by 0.001 led to increased differences between 4D-CS and 4D-PI of −0.4% (*P* = 0.0021) in healthy participants. There were significant differences for ventricular kinetic energy (systole: *P* < 0.0001; diastole: *P* < 0.0001) and haemodynamic forces (systole: *P* < 0.0001; diastole: *P* < 0.0001), where error increased with increasing *λ* values in both healthy participants and patients.

**Conclusion:**

4D flow CMR with CS can be used clinically to assess SV in paediatric and adult patients. Ventricular kinetic energy and haemodynamic forces are however sensitive to the change in reconstruction parameter *λ*, and it is therefore important to validate advanced blood flow measurements before comparing data between scanners and centres.

## Graphical Abstract

**Graphical Abstract:**
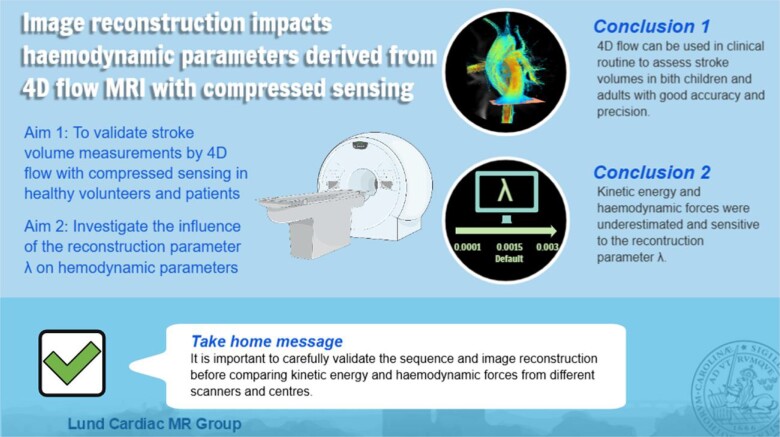

## Introduction

Blood flow measurements are central to cardiac disease evaluation, especially in valvular disease and congenital heart defects (CHDs).^1,2^ Cardiac magnetic resonance imaging (CMR) applies 2D phase-contrast flow measurements with high accuracy and precision.^3^ In complex CHD, however, several flow measurements are often needed, requiring highly skilled CMR technologists with special knowledge in CHD and their diverse anatomies to plan every flow measurement.^4^ Many 2D flow assessments also lead to long examinations.^5^ 3D time-resolved 4D flow measurements can potentially shorten the examination time by providing flow measurements for multiple vessels and across valves simultaneously without the need for breath-holding. During post-processing, blood flow measurements can be performed in all vessels within the examined volume and there is also the option of valve tracking.^6,7^ Avoiding extensive CMR examinations is preferable, particularly for young children, regardless of whether the patient is awake, sedated, or under general anaesthesia. A previous study on neonates, where anatomy and flow volumes in five vessels were assessed, showed that 4D flow measurements were almost three times faster than multiple 2D flow measurements,^8^ making further development of the method of great interest. 4D flow CMR may also add diagnostic, and possibly prognostic, information through the ability to measure advanced haemodynamic parameters like kinetic energy and haemodynamic forces.^8^

However, low spatial and temporal resolution means that this method has limited clinical use, especially in neonates and small children.^9,10^ Compressed sensing (CS) is an image reconstruction method used for 4D flow (4D-CS) blood flow measurements^11-13^ that can provide acceleration of scan time and/or provide higher spatiotemporal resolution. 4D-CS has been shown to reconstruct images with higher resolution and shorter scan time^14^ than conventional acceleration using parallel imaging (4D-PI). In short, 4D-PI uses the different coverage areas of the multiple receiver coil elements to accelerate imaging. For 4D-CS acceleration, additional a priori knowledge about the images is added, e.g. that consecutive time frames over the heartbeat share information. Since additional knowledge about the images is accounted for, imaging can be further accelerated. It is however unknown whether 4D-CS can be used to compute advanced haemodynamic measures such as ventricular kinetic energy^15-17^ and haemodynamic forces^18-21^—measures that have been suggested as biomarkers for cardiac function. Prior to clinical application, it needs to be confirmed that data based on 4D-CS CMR provide accurate measurements of these advanced measures.

Furthermore, 4D-CS image reconstruction is dependent on the parameter *λ*, which is used to tune how strictly a priori knowledge is enforced in the reconstructed image. A high value of *λ* means that the a priori knowledge is given high importance, and a lower value of *λ* means that consistency with the acquired data is emphasized. The choice of *λ* must be high enough to enable a high-quality image reconstruction without noise and under-sampling artefacts, but if *λ* is too high, the image will be over-smoothed, resulting in a lower spatial resolution and reduced accuracy of flow measurements. However, it is unknown how the choice of *λ* impacts measurements of stroke volumes (SVs) and the advanced 4D flow measures of ventricular kinetic energy and haemodynamic forces. As implementation of 4D-CS image reconstruction differs between sites, the impact of *λ* is crucial in understanding results from different studies and important for CHD multicentre studies using 4D-CS flow data.

Therefore, the aims of the study were to (i) validate SV by 4D-CS to SV by 4D-PI and by 2D flow; (ii) validate 4D-CS for clinical use by comparing SV by 4D-CS to SV by 2D flow in paediatric and adult patients; and (iii) assess the influence of the 4D-CS reconstruction parameter *λ* on ventricular kinetic energy and haemodynamic forces.

## Methods

### Study population

Healthy adult participants (*n* = 10) without known cardiac disease or medication were prospectively recruited for CMR. Patients (*n* = 30) referred for clinical assessment with CMR including flow measurements were prospectively and non-randomly recruited in chronological order of examination. If the clinical schedule allowed the extra examination time, these patients were asked to participate in the study and if so 4D-CS was added to the clinical CMR. Only 4D-CS was acquired in patients, in contrast to volunteers who had both 4D-CS and 4D-PI obtained. Exclusion criteria were contraindications to CMR.

### Imaging methods and parameters

Examinations were performed using a 1.5 Tesla MAGNETOM Aera (Siemens Healthineers, Erlangen, Germany). A posterior coil array integrated into the patient table was used in combination with an 18-channel anterior coil array. A 4D-CS flow research prototype sequence with a Cartesian spiral phyllotaxis pattern was used, with spatially and temporally incoherent variable density sampling.^22^ Image reconstruction was performed using a standard CS non-linear iterative method.^23^

In healthy participants, three different flow sequences were applied: (i) 2D flow; (ii) the above 4D-CS sequence^22^; and (iii) a conventional Cartesian 4D flow research prototype sequence with PI acceleration implemented using generalized autocalibrating partially parallel acquisitions (GRAPPA). *Table 1* shows sequence parameters, and *Table 2* shows scanning protocols for healthy volunteers. The 2D flows were acquired in the ascending aorta, descending aorta, superior vena cava, pulmonary artery, and lower right pulmonary vein. To assess cardiac output variability in each individual, 2D flow was acquired in the ascending aorta both before and after 4D flow acquisitions. Also, to avoid possible bias due to variability over examination time, 4D flow acquisitions were performed in random order. Finally, to test interscan variability, six volunteers had the 4D-CS sequence repeated after a short break outside the scanner. A short-axis balanced steady-state free-precession (bSSFP) cine stack covering the entire heart was acquired for ventricular delineations to assess ventricular kinetic energy and haemodynamic forces.

**Table 1:** Sequence parameters

Acquisition parameters	4D flow compressed sensing	4D flow parallel imaging	2D flow
Field of view (read × phase × slice, mm)	Whole heart 240 × 240 × 140	Whole heart 288 × 288 × 144	320 × 158 × 5 mm
Spatial resolution (acquired and reconstructed, read × phase × slice, mm)	2.5 × 2.5 × 2.5	3 × 3 × 3	1.5 × 1.5 × 5
Slab/slice orientation	Sagittal	Sagittal	Transversal (aorta), oblique (pulmonary artery)
Effective temporal resolution (acquired and reconstructed, ms)	38.8	38.6	9.8
Temporal segmentation factor	2	2	1
ECG synchronization	Retrospective	Retrospective	Retrospective
Respiratory motion compensation	Navigator on liver/diaphragm, 6 mm window size	Navigator on liver/diaphragm, 6 mm window size	None
TR/TE/flip (ms/ms/°)	2.32/4.85/8	2.3/4.83/7	2.7/4.9/20
Acceleration methods	Compressed sensing	GRAPPA *R* = 2 in anterior–posterior direction and partial Fourier factor 6/8 in phase and slice directions	
Net acceleration factor	7.7	7.1	
VENC, cm/s	150	150	200
Postprocessing parameters	Maxwell correction (4D flow), background correction using static tissue, phase unwrapping if needed.		

ECG, electrocardiogram; TR, repetition time; TE, echo time; GRAPPA, generalized autocalibrating partially parallel acquisitions; VENC, velocity encoding.

**Table 2:** Scan protocol for healthy participants

Sequence order	Description
1	Localizer images
2	Cine images (short axis, long axis)
3	2D flow of ascending and descending aorta, pulmonary artery, superior vena cava, and lower right pulmonary vein
4	4D-CS and 4D-PI (in random order)
5	2D flow ascending aorta
6	Break (participant removed from table, then repositioned)
7	Localizer images
8	2D flow ascending aorta
9	4D-CS

CS compressed sensing; PI, parallel imaging.

Patients underwent the protocol relevant for the clinical question and then a 4D-CS sequence was acquired.

The 4D-CS images were reconstructed online on the scanner by iterative solution of the optimization problem.^22^


$$\{x_{t}{\}}_{t=1,\ldots ,T}=argmin_{\left\{x_{t}\right\}}\sum_{t=1}^{T}\left(\|A_{t}x_{t}-y_{t}{\|}_{2}^{2}+\lambda {\left\|W_{\sigma }x_{t}\right\|}_{1}\right)+K\lambda \|W_{\tau }\{x_{1}^{T},\ldots ,x_{T}^{T}\}{\|}_{1}.$$

Here **x***_t_* denotes the reconstructed images for each timeframe *t* = 1…, *T*; **A***_t_* is the measurement forward operator for timeframe *t*; and **y***_t_* is the data for timeframe *t*. The operator **W**_σ_ performs a spatial wavelet transform, and **W**_τ_ a temporal finite difference transform. The factor *λ* governs the strength of the CS regularization with a default value of *λ* = 0.0015 with data normalized such that the maximum image intensity is 1, and *K* provides a balancing factor between spatial and temporal regularization set to *K* = 5.^22^ The number of iterations was set to 30 for all reconstructions.

Furthermore, offline retrospective reconstructions were performed in the six healthy volunteers and six patients for additional *λ* values of 0.0001, 0.0005, 0.001, 0.002, and 0.003, to assess the impact of *λ* on the calculation of SV, ventricular kinetic energy, and haemodynamic forces. The values of *λ* were chosen to vary by a factor of 30 between the lowest and highest around the previously used value of *λ* = 0.0015,^22^ a slightly larger range compared to previous work by Braig *et al.*^24^

### Flow analysis

The software CAAS MR Solutions 5.1.1 (Pie Medical Imaging, Maastricht, The Netherlands) was used to measure 4D SV. Segment (Medviso AB, Lund, Sweden) was used to measure 2D SV and analyse ventricular kinetic energy, haemodynamic forces, and the impact of reconstruction parameter *λ* values. First-order linear phase background correction was performed in all flow data. Visually, no phase wrapping was observed in any of the data sets, and therefore, phase unwrapping was not necessary.

One observer with 10-year CMR experience (PS) analysed all data. For assessment of interobserver variability, another observer with 1.5-year CMR experience (JW) analysed flow in the healthy participants.

In the healthy participants, SVs assessed by 2D flow, 4D-CS, and 4D-CPI were compared in the ascending aorta, descending aorta, main pulmonary artery, and superior vena cava. In patients, SVs assessed by 2D flow and 4D-CS were compared for the vessels where 2D flow was available—typically for the ascending aorta, descending aorta, main pulmonary artery, left pulmonary branch, right pulmonary branch, superior vena cava, and inferior vena cava. For assessment of repeatability, 2D and 4D-CS scans were repeated after a short break outside the scanner. For assessment of 4D flow internal consistency, flow differences between the ascending aorta and the main pulmonary artery were used, except in patients with a single ventricle. For patients in whom cine images to assist valve tracking were available, 4D flow internal consistency was also assessed by comparing the SV through the ascending aorta or main pulmonary artery to that through the corresponding atrioventricular valve.

### Statistical analysis

Statistical analyses were performed using GraphPad (GraphPad Software, Boston, MA, USA, www.graphpad.com) and Excel (Microsoft Corporation). Data are reported as median and range or interquartile range. Differences in scan time were assessed by the Mann–Whitney test. Interobserver reliability was estimated by Bland–Altman plots with 95% limits of agreement (in absolute and proportional bias) intraclass correlation coefficient (ICC).^25,26^ Test–retest agreement was estimated by Bland–Altman plots with 95% limits of agreement (in absolute and proportional bias) and by using the repeatability coefficient.^25^ Internal consistency and agreement in SV were assessed by Bland–Altman plots with 95% limits of agreement (in absolute and proportional bias) and ICC. ICC < 0.5 was interpreted as poor reliability, 0.5–0.75 as moderate, 0.75–0.9 as good, and >0.9 as excellent reliability.^27^ Trends in the reconstruction factor *λ*’s impact on SV, ventricular kinetic energy, and haemodynamic forces were assessed using a linear mixed model with the slope as fixed effect and random intercept for each subject, using the function ‘fitlme’ in the Statistics and Machine Learning Toolbox in Matlab R2022a (Mathworks, Natick, MA, USA). Statistical significance for the fixed effect was assessed with analysis of variance using the Satterthwaite approximation for degrees of freedom.^28^

## Results

### Healthy participants

Ten healthy participants were scanned. For one of them, the 4D-CS data were not possible to import into the analysis software and they had to be excluded. Median age was 29 (20–62) years, 4 men and 5 women. There was no difference in average scan time between 4D-CS [10:59 (5:21–14:14) min with a 62% navigator efficiency; *P* = 0.21] and 4D-PI [9:31 (6:59–11:34) min with a 61% navigator efficiency].

The bias at repeated measurements in the ascending aorta before and after the short break was for 2D flow −1.1 ± 5.2 mL (proportional bias −1.2 ± 5.4%; repeatability coefficient 14.4 mL) and for 4D-CS 0.0 ± 5.5 mL (0.1 ± 8.3%; repeatability coefficient 15.2 mL).

Interobserver agreement and reliability for SV by 2D acquisitions was 0.5 ± 2.6 mL (1.0 ± 5.3%; ICC 0.97), by 4D-PI was 0.8 ± 4.4 mL (2.3 ± 7.3%), and by 4D-CS was 1.1 ± 2.5 mL (2.3 ± 5.6%; ICC 0.96).

**Figure 1:**
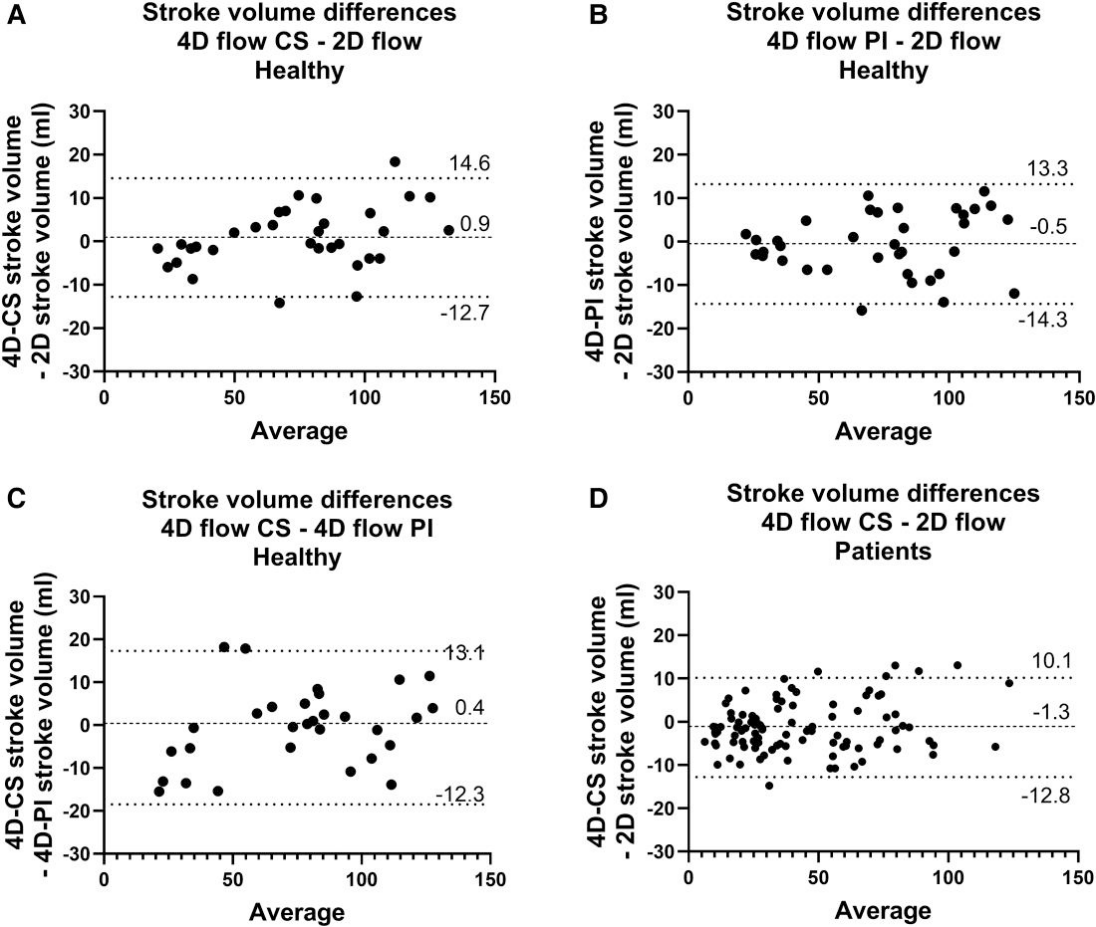
SV in healthy participants. Bland–Altman plots show differences between SV assessed by (*A*) 2D flow and 4D-CS, (*B*) 2D flow and 4D-PI volumes, and (*C*) 4D-CS and 4D-PI. Both 4D-CS and 4D-PI agree with 2D flow to a similar extent as repeated 2D or 4D flow acquisitions (cf. Results section). (*D*) Bland–Altman plot showing difference in SV between 2D flow and 4D flow with CS in 29 patients, and a total of 97 vessels.

*Figure 1A–C* show agreement in SV between the three flow sequences. SV by 4D-CS and 2D flow differed by 0.9 ± 7.0 mL (0.9 ± 10.6%; ICC 0.98) and SV by 4D-PI and 2D flow differed by −0.5 ± 7.0 mL (−1.6 ± 9.3%; ICC 0.97). Also, 4D-CS SV differed from 4D-PI SV by 0.4 ± 6.5 mL (0.6 ± 9.1%; ICC 0.98).

Internal consistency analysis showed that 4D-CS SV in the ascending aorta differed from SV in the pulmonary artery by −0.5 ± 3.8 mL (−0.4 ± 4.0%; ICC 0.98) and 4D-PI SV in the ascending aorta differed from SV in the pulmonary artery −0.5 ± 6.1 mL (−0.4 ± 5.8%; ICC 0.95).

### Patients

Thirty patients were scanned but one patient was excluded due to corrupt 4D flow data set. Median age was 16 (2–75) years, 21 men and 8 women. Twenty-one patients were younger than 18 years, and 17 patients had some form of CHD of which 9 had single ventricles. In total, 97 vessels were analysed with both 2D flow and 4D-CS. Mean scan time for 4D-CS in patients was 10:33 min with 66% navigator efficiency (*n* = 27, no information could be retrieved for 2 of the patients).

SV assessed by 2D flow differed from 4D-CS SV by 1.3 ± 6.0 mL (7.2 ± 20%, ICC 0.97) (*Figure 1D*). Internal consistency analysis for 4D-CS showed a difference between SV in the ascending aorta and in the pulmonary artery of −0.6 ± 5.9 mL (−1.0 ± 10.0%, ICC 0.97, *n* = 21), a difference between right ventricular inflow SV and outflow SV of −0.9 ± 7.5 mL (−1.0 ± 10.2%, ICC 0.90, *n* = 17), and a difference between left ventricular (or systemic ventricular in cases of single ventricles) inflow SV and outflow SV of −0.2 ± 5.8 mL (−0.1 ± 13.3%, ICC 0.97, *n* = 23).

### Ventricular kinetic energy and haemodynamic forces

Peak left ventricular kinetic energy in healthy volunteers differed between 4D-CS with default reconstruction factor *λ* = 0.0015 and 4D-PI both during systole (−0.7 ± 0.7 mJ, −15 ± 16%; ICC 0.85) and during diastole (−1.5 ± 0.8 mJ, −32 ± 15%; ICC 0.72). Likewise, ventricular haemodynamic forces differed between 4D-CS with default *λ* = 0.0015 and 4D-PI both during systole (−0.11 ± 0.09 N, −20 ± 14%; ICC 0.60) and during diastole (-0.11 ± 0.05, −30 ± 14%; ICC 0.55).

### Impact of reconstruction parameter *λ*

**Figure 2:**
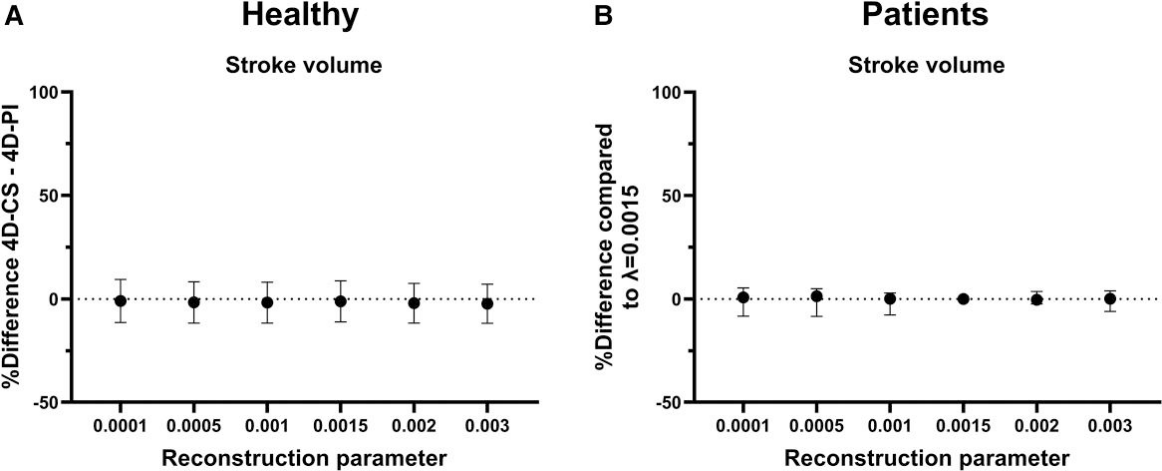
Impact of change in reconstruction parameter on SV in the ascending aorta. (*A*) Comparison of 4D-CS and 4D-PI and different reconstruction parameters in eight healthy volunteers and (*B*) 4D-CS with default setting (*λ* = 0.0015) and different reconstruction parameters in six patients. Values are presented as median and interquartile range. The agreement between 4D-CS and 4D-PI was not affected by change in reconstruction parameter.

**Figure 3:**
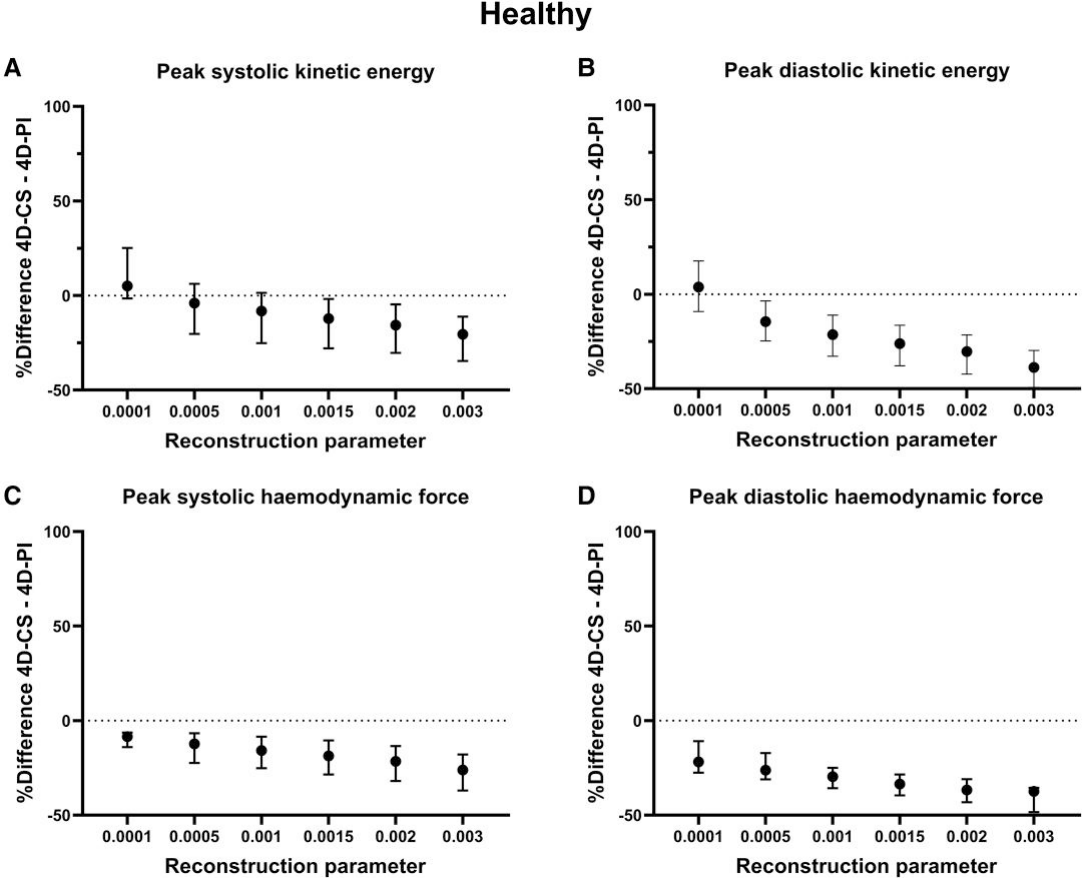
Impact of change in reconstruction parameter on ventricular kinetic energy and haemodynamic forces in healthy volunteers (*n* = 6). Difference in peak ventricular kinetic energy (*A* and *B*) and haemodynamic forces (*C* and *D*) in the left ventricle during systole (*A* and *C*) and diastole (*B* and *D*) between 4D-CS and 4D-PI with different reconstruction parameters. Values are presented as median and interquartile range. Both ventricular kinetic energy and haemodynamic forces were lower in 4D-CS than in 4D-PI with default reconstruction parameter (*λ* = 0.0015). The reconstruction parameter *λ* has a significant impact on the parameters.

**Figure 4:**
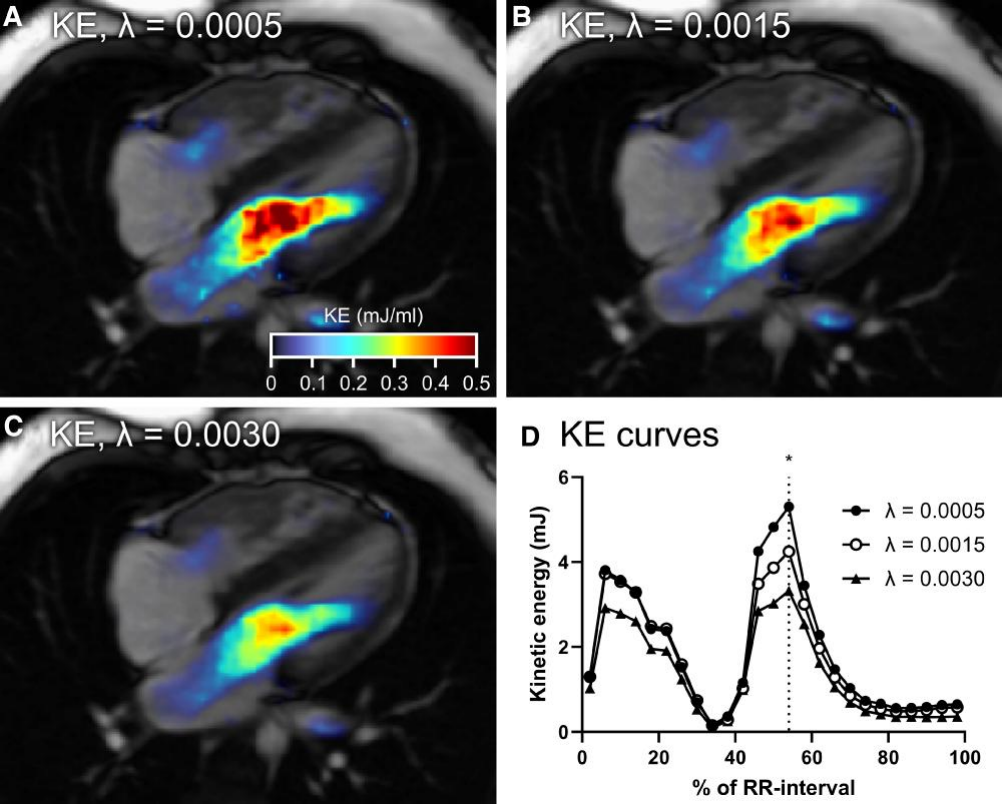
Visualization of ventricular kinetic energy (KE) in the left ventricle during early diastole in a patient. Images show peak KE with (*A*) *λ* = 0.0005, (*B*) *λ* = 0.0015, and (*C*) *λ* = 0.0030. Peak KE decreased with as *λ* increased. (*D*) KE across the cardiac cycle in the same patient with different reconstruction parameters. The asterisk and vertical dashed line show the time frame of peak diastolic KE where the images were generated.

*Figure 2* shows that the reconstruction factor value impacted SV measurements in healthy subjects (−0.4% per 0.001 increase in *λ*, *P* = 0.0021) but not in patients (0.3% per 0.001 increase in *λ*, *P* = 0.75). Although statistically significantly different, this has no clinical significance. *Figure 3* shows that increasing reconstruction factor values resulted in increasing differences between 4D-CS and 4D-PI for both peak ventricular kinetic energy in systole (−10% per 0.001 increase in *λ*, *P* < 0.0001) and diastole (−13% per 0.001 increase in *λ*, *P* < 0.0001) as well as for haemodynamic forces in systole (−6% per 0.001 increase in *λ*, *P* < 0.0001) and diastole (−8% per 0.001 increase in *λ*, *P* < 0.0001). *Figure 4* visualizes the effect of *λ* on ventricular kinetic energy in a patient during early diastole.

**Figure 5:**
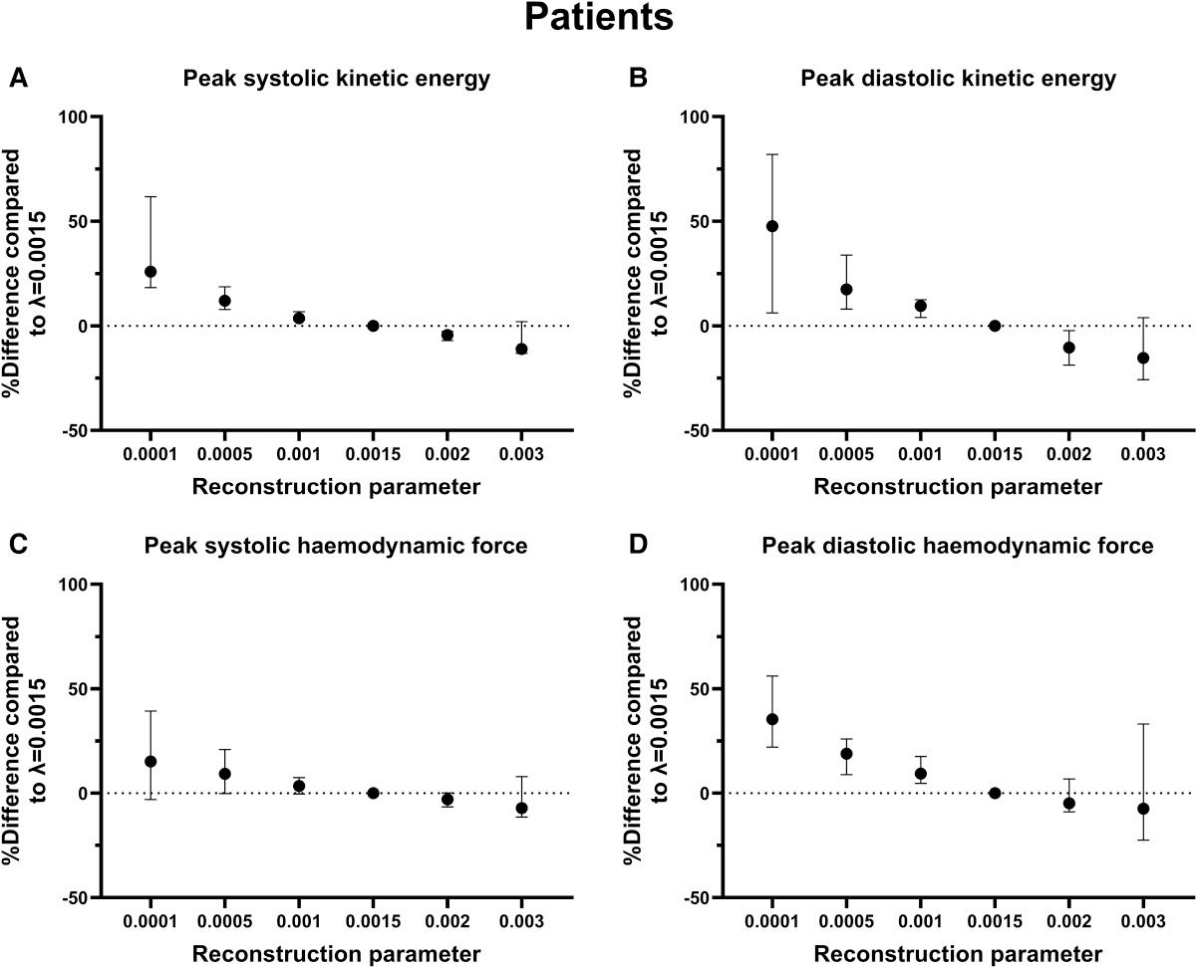
Impact of change in reconstruction parameter on ventricular kinetic energy and haemodynamic forces in patients (*n* = 6). (*A*) and (*B*) show the difference in peak ventricular kinetic energy, and (*C*) and (*D*) show differences in haemodynamic forces in the left ventricle between 4D-CS with default setting (reconstruction parameter *λ* = 0.0015) and 4D-CS with different reconstruction parameters, in six patients. (*A*) and (*C*) show systolic values, and (*C*) and (*D*) show diastolic values. Values are presented as median and interquartile range. The reconstruction parameter *λ* has a significant impact on the values for both ventricular kinetic energy and haemodynamic forces.

**Figure 6:**
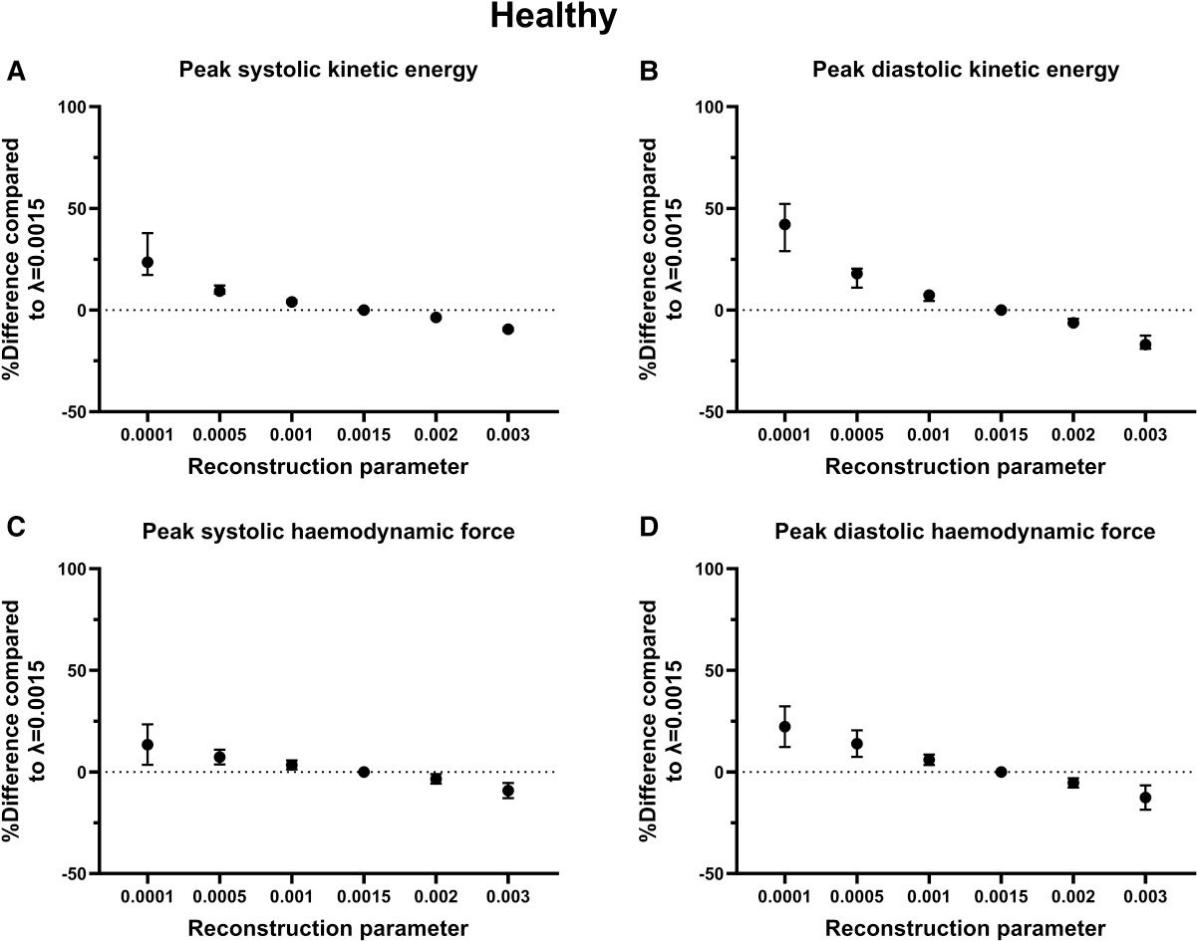
Impact of change in reconstruction parameter on ventricular kinetic energy and haemodynamic forces in healthy participants (*n* = 8). Difference in peak ventricular kinetic energy (*A* and *B*) and haemodynamic forces (*C* and *D*) in the left ventricle during systole (*A* and *C*) and diastole (*B* and *D*) between 4D-CS and default setting (reconstruction parameter *λ* = 0.0015) and 4D-CS with different reconstruction parameters. Values are presented as median and interquartile range. The reconstruction parameter *λ* has a significant impact on the values for both ventricular kinetic energy and haemodynamic forces.

Reference 4D-PI acquisitions were not available in patients, and so peak ventricular kinetic energy and haemodynamic forces were compared to default value of *λ*. Increased *λ* resulted in lower peak kinetic ventricular kinetic energy in systole (−14% per 0.001 increase in *λ*, *P* < 0.001) and diastole (−19% per 0.001 increase in *λ*, *P* < 0.0001) and haemodynamic forces in systole (−7% per 0.001 increase in *λ*, *P* = 0.016) and diastole (−10% per 0.001 increase in *λ*, *P* = 0.0013) (*Figure 5*). Similar trends (*Figure 6*) were seen in healthy subjects for ventricular kinetic energy in systole (−11% per 0.001 increase in *λ*, *P* < 0.0001) and diastole (−18% per 0.001 increase in *λ*, *P* < 0.0001) and haemodynamic forces in systole (−8% per 0.001 increase in *λ*, *P* < 0.0001) and diastole (−12% per 0.001 increase in *λ*, *P* < 0.0001).

## Discussion

This study shows that 4D flow CMR with CS can be used clinically to accurately and precisely and with internal consistency assess SV in both children and adults, including patients with CHD. However, ventricular kinetic energy and haemodynamic forces varied substantially with different values for *λ* in both healthy participants and patients, highlighting the importance of validating the sequences used before comparing data between scanners.

Differences between 4D and 2D SV as well as 4D-CS and 4D-PI SV were small, and similar to differences between repeated 2D and repeated 4D acquisitions, and to differences between observers (interobserver variation). Thus, differences in SV between 4D and 2D flow measurements could at least in part be explained by measurement variability and short-term variations in physiology. The current results are in line with earlier studies using 4D flow both with CS^13,29,30^ and PI.^31^ This indicates that 4D-CS can be used clinically for SV measurements. The current study, however, also extends knowledge by including assessment of ventricular kinetic energy and haemodynamic forces. This study also demonstrates the wide applicability of 4D-CS by including patients with CHD and also a wide range of ages (2–75 years) to include small vessels.

The current study shows that 4D-CS is well suited for assessment of valvular disease or shunts based on SV measurements. This has been reported previously.^10,12,29^ Again, the current study also shows the wide clinical utility by including patients with a large age span who were examined as part of the ordinary clinical practice and thus by different technicians with various level of experience.

Ventricular kinetic energy and haemodynamic forces are measurements that can be derived from 4D flow data and are gaining interest as biomarkers for cardiac dysfunction.^15,16,18–21,32–34^ Ventricular kinetic energy is the work needed to accelerate the blood from rest to the stated velocity. Disturbed filling and emptying of the ventricles alters the kinetic energy in patients with heart failure,^34^ after myocardial infarction^35^ and in patients with CHDs such as Tetralogy of Fallot^15,33^ and Fontan circulation.^17^ Haemodynamic forces are caused by intraventricular pressure gradients that are formed when the ventricle contracts and relaxes, causing the blood to accelerate. Changes in contraction and relaxation patterns affect these forces, and haemodynamic forces may be a more sensitive marker of altered cardiac function than volumes and ejection fraction.^36^ These parameters could potentially be used to find early signs of impaired cardiac function to intervene and avoid future heart failure.

The CHD patient populations are small, and anatomy and physiology often heterogenous within the same heart defect,^37^ which means that single-centre studies may lead to underpowered studies and risk missing important results. Multicentre studies are becoming more common, and it is important to understand how differences in vendor hardware implementations, sequences, and reconstruction methods affect measurements based on 4D flow data if you want to compare or pool data from different centres or scanners. Before starting a multicentre study with pooled data, measurement agreement for data from advanced flow measurements should be assessed between centres, e.g. by comparing phantom data^38,39^ or by examining the same volunteers in all centres.

Both ventricular kinetic energy and haemodynamic forces based on 4D-PI are validated by laser particle image velocimetry in phantoms,^18,31^ whereas validation is lacking for 4D-CS. The current study shows that ventricular kinetic energy and haemodynamic forces are underestimated by 4D-CS compared to 4D-PI. However, there was some improvement when the reconstruction factor *λ* was set to lower values for image reconstruction. This further points to the fact that every step from acquisition to reconstruction parameters needs to be considered when comparing data from different sites in multicentre studies when including ventricular kinetic energy or haemodynamic forces computed from 4D-CS data. Our results are in line with similar data from Braig *et al.*^24^ who used 4D-CS in a small animal model and found that peak velocities are influenced by the choice of *λ*. Care should also be taken when assessing other advanced parameters derived from 4D-CS, such as wall shear stress, energy loss, vorticity, and helicity of flows. While SV quantification was less sensitive to *λ*, validation and quality control should still be performed to ensure good accuracy and precision as other details of image acquisition and reconstruction could influence results. From a technical perspective, research into image reconstruction methods for accelerated MRI that are robust to changes in reconstruction parameters such as *λ* is warranted to potentially avoid this source of variability in the future.

### Limitations

There was no external validation in this study. However, flow volumes from 2D flow CMR, which is a validated method,^3^ was used as the gold standard. Also, kinetic energy using 4D-PI has been validated previously,^31^ as has haemodynamic forces.^18^

## Conclusion

4D flow CMR with CS can be used in clinical routine to assess SV in both children and adults, including patients with CHD, and with good accuracy, precision, and internal consistency. Ventricular kinetic energy and haemodynamic forces were underestimated and sensitive to the reconstruction parameter *λ*, emphasizing the importance of careful validation of the sequence and image reconstruction before comparing such data from different scanners and centres.

## Data Availability

The data sets in this article are not readily available because of patient data privacy concerns. Other data will be made available upon reasonable request and should be directed to pia.sjoberg@med.lu.se.

## Lead author biography

Pia Sjöberg is a specialist in cardiothoracic surgery and clinical physiology working at Skåne University Hospital in Lund, Sweden. Her research focus is congenital heart defects, using novel MRI techniques, 4D flow, and personalized computational modelling to improve the understanding of heart failure and to predict the results of interventions.
